# Analysis of the Security and Privacy Requirements of Cloud-Based Electronic Health Records Systems

**DOI:** 10.2196/jmir.2494

**Published:** 2013-08-21

**Authors:** Joel JPC Rodrigues, Isabel de la Torre, Gonzalo Fernández, Miguel López-Coronado

**Affiliations:** ^1^Instituto de Telecomunicações, University of Beira InteriorCovilhaPortugal; ^2^University of ValladolidValladolidSpain

**Keywords:** cloud-computing, eHealth, electronic health records (EHRs), privacy, security

## Abstract

**Background:**

The Cloud Computing paradigm offers eHealth systems the opportunity to enhance the features and functionality that they offer. However, moving patients’ medical information to the Cloud implies several risks in terms of the security and privacy of sensitive health records. In this paper, the risks of hosting Electronic Health Records (EHRs) on the servers of third-party Cloud service providers are reviewed. To protect the confidentiality of patient information and facilitate the process, some suggestions for health care providers are made. Moreover, security issues that Cloud service providers should address in their platforms are considered.

**Objective:**

To show that, before moving patient health records to the Cloud, security and privacy concerns must be considered by both health care providers and Cloud service providers. Security requirements of a generic Cloud service provider are analyzed.

**Methods:**

To study the latest in Cloud-based computing solutions, bibliographic material was obtained mainly from Medline sources. Furthermore, direct contact was made with several Cloud service providers.

**Results:**

Some of the security issues that should be considered by both Cloud service providers and their health care customers are role-based access, network security mechanisms, data encryption, digital signatures, and access monitoring. Furthermore, to guarantee the safety of the information and comply with privacy policies, the Cloud service provider must be compliant with various certifications and third-party requirements, such as SAS70 Type II, PCI DSS Level 1, ISO 27001, and the US Federal Information Security Management Act (FISMA).

**Conclusions:**

Storing sensitive information such as EHRs in the Cloud means that precautions must be taken to ensure the safety and confidentiality of the data. A relationship built on trust with the Cloud service provider is essential to ensure a transparent process. Cloud service providers must make certain that all security mechanisms are in place to avoid unauthorized access and data breaches. Patients must be kept informed about how their data are being managed.

## Introduction

Cloud computing environments provide a great opportunity to provide eHealth services in different scenarios in an effective and simple way. The scalability and mobility that a Cloud-based environment system can offer provides several advantages [1-9], but there are some barriers that must also be managed [10,11]. In the case of deploying a Cloud-based EHR management system, the main advantage is the ability to share patient records with other clinical centers, and the integration of all the EHRs of a group of clinical centers in order to help medical staff perform their jobs [12-14]. So, how can health care providers and clinical centers guarantee the security, privacy, and confidentiality of their patients’ data? The privacy and security of data migrated to the Cloud represents the main barrier that the Cloud computing paradigm must overcome if a Cloud-based eHealth environment is to be deployed. This mission must be performed by both Cloud service providers and health care providers, since hosting EHRs in the Cloud requires a change of approach and they must take into account and address all these risks [15-17].

Security issues are critical when a health care provider plans to deploy a Cloud-based EHR management system. The health care provider must guarantee the security of patient data by ensuring that the Cloud platform has the needed security mechanisms in place. Transmission and network secure protocols also must be deployed in order to avoid external attacks to the data [18]. Moving patient data to the Cloud means that patient files are hosted in the servers of the Cloud service provider [19]. What does this mean? It is essential that these companies ensure the security of their databases so that the data cannot be accessed or modified by unauthorized users. It is important to be aware that privacy and confidentiality terms are essential when EHRs are migrated to the Cloud because of the sensitivity of patient data. In order to avoid unauthorized access, Cloud service providers must deploy authentication systems that ensure the privacy of patient information.

Governments must require that Cloud service providers fulfill the privacy requirements needed to ensure the privacy of patient data. The deployment of a legal framework will help to accomplish a secure environment [13,14]. Privacy policies have been legislated in several countries in order to regulate and safeguard the privacy of patient records. As an example, the US Health Insurance Portability and Accountability Act (HIPAA) regulates the privacy and security of US patient data [20]. These policies depend on each country. Furthermore, EHRs themselves are ruled by standards, which include security and privacy terms, such as Health Level 7 (HL7) [21,22], to guarantee data security and privacy. By combining these standards with Cloud policies and security mechanisms implemented by providers, a secure “Health Cloud” scenario will be achieved.

This paper addresses the health care providers’ security and privacy issues that must be considered when deploying EHR management systems. Taking into account these issues on both sides, the migration process will be more secure and transparent. Some security mechanisms necessary to deploy a proper solution are suggested.

We will first elaborate on the issues and requirements for maintaining the security and privacy of EHRs. After that, we explain the requirements that a Cloud-based EHR management system must guarantee in terms of security. Also, some suggestions are given to health care providers in order to facilitate the process.

## Methods

For the analysis and study of Cloud-based EHR systems, we reviewed published papers and research about security and privacy issues, which different Cloud computing providers use for development of their Cloud platforms. The related literature was obtained mainly from Medline sources. Direct contact with some Cloud service providers was made. Many publications that show the feasibility of Cloud computing implementations for eHealth services were reviewed in order to look for the latest information on this emerging technology. Most of them show the advantages that Cloud-based solutions can provide to eHealth systems.

## Results

### Electronic Health Record Security and Privacy Issues

The deployment of EHR management systems is one of the most important achievements in eHealth in recent years. The implementation of these systems has been growing rapidly. In fact, most developed countries have a high level of penetration of this kind of system.

According to Spanish law 41/2002, an EHR is defined as the documentation, which contains information about the clinical evolution of the patient during his or her health assistance process. In this law, the uses of EHRs are set out, requiring medical personnel to maintain the privacy of patients. The Spanish law treats this kind of information as “specially protected” files. This kind of nomenclature is set in the 15/1999 law with the purpose of guarding the privacy of sensitive patient information. The patient’s consent is required to manage and access this data, except in the case of an emergency where the patient’s life is at risk.

In the United States, HIPAA regulates and establishes the security and privacy requirements of patient data. This law includes two sections on avoiding the improper use of personal information: the Privacy Rule and the Security Rule. The HIPAA Privacy Rule establishes that the Protected Health Information (PHI) must be made available in order to provide the patient medical treatment, either with a Court order or with the authorization of the patient. This rule adds that the entities that use the health information must notify the patient about the use of their PHI. Furthermore, the Privacy Rule requires that entities accessing the PHI use the least amount of patient data necessary to meet their needs. The HIPAA Security Rule was set in 2003 and complements the Privacy Rule, adding several terms to address the digitalization of the patient health information. It has three kinds of security guarantees: administrative, technical, and physical [23-25].

Thus, as outlined above, health care providers must guarantee and preserve the security and privacy of EHRs, and then implement the required security mechanisms to keep patient information safe in the Cloud. Before explaining the mechanisms that a Cloud service provider must implement, we describe the security and privacy requirements of patient records.

### Electronic Health Record Security and Privacy Requirements

Before moving EHRs to the Cloud, the EHR systems themselves must set several guarantees to preserve sensitive patient information. The combination of these security requirements with those of the Cloud systems will guarantee the privacy and security of EHRs hosted in the Cloud. The requirements to secure an EHR are described in Table 1 [22]. The security and privacy issues that a Cloud-based system must address in order to safeguard patient files are analyzed in the next section.

### Security and Privacy Issues of Cloud-Based Health Solutions

Deploying Cloud-based health solutions is an important step in the development of eHealth. Cloud-based systems allow the ability to create scalable environments, which are adapted to user needs. This total adaptation is complemented by the savings offered by a pay-per-use system, like Cloud computing. Another great advantage comes from the fact that, when EHRs are hosted in the Cloud, medical personnel or patients have the ability to access the information at any time from wherever they have an Internet connection. Currently, with the global economic crisis, saving money could be one of the most important reasons that would drive a company to move its electronic health system into the Cloud. Therefore, Cloud service providers must take advantage of this fact when selling their prospective clients on the advantages of Cloud-based systems.

In order to guarantee the security of their systems, Cloud service providers must install several security mechanisms to keep the safety, privacy, and security of their clients’ data. In the section below, we explain the different mechanisms that a Cloud service provider implements in its systems to maintain the security of files in the context of EHR security.

#### eHealth Cloud Security Issues

A Cloud-based EHR must maintain the same level of data security as data stored in the servers of the health care provider. Patients and medical personnel should know that their personal information is going to be stored with a third-party provider; the provider must guarantee the same security and privacy that the EHRs had in the local servers. The patient, obviously, is not involved in the process of moving their sensitive information to the Cloud, but information should be communicated to patients by the health care providers about the data migration. These communications are not simple notifications; instead, patients should be informed about all the advantages that a Cloud-based system offers for the management of their medical information. Patients should know that data management responsibility lies with both parties: the Cloud service provider and, in a more active way, the health care provider or clinical center. However, there are security issues that should be considered by both providers and customers of a Cloud-based EHR system.

**Table 1:** Requirements for maintaining the security and privacy of an electronic health record.

Requirements	Description
Authorized access	In order to deploy an authorized-control system, it is essential to deploy an identification system for both patients and health care providers. This identification must be portable between the different entities that have access to the patients’ data. This system might be achieved by the ID identifier of each patient. Regarding the authentication, a centralized system based on a public key is viable. A RBAC (Role-Based Access Control) should be deployed in order to allow authorized personnel access to specific data based on their role.
Confidentiality	To guarantee the confidentiality of the communication process, encryption algorithms are used. However, the confidentiality problem in a distributed system arises because it is not possible for the information transmitter system to verify that confidentiality has not been exposed on the receiving end.
Patient’s consent	According to the legislation, patients must allow or deny access to their clinical information, except in emergency situations. This consent could be implicit or explicit. Another fact to consider is the need to get access to the EHR-hosted entity from another external one. This process should have the consent of the patient, but in case of emergency, a security mechanism must be provided to avoid this restriction without the patient’s consent.
Relevance	All the medical personnel who take part in the diagnostic and treatment process have access to the EHR. Administrative personnel will be able to access the clinical information if their function is relevant to the medical process. Therefore, only the relevant personnel will get access to the patient information. To guarantee that only this level of personnel has been able to access the data, an access control system must be deployed. Given the difficulty of establishing information relevance, it is preferable to have a default permission access and, if necessary, study possible abuses.
Information ownership	The ownership of the EHR is not clearly established. The medical personnel are responsible for this information. However, the patients themselves have the right to access their clinical information.
Information consistency	In an interoperability outline, a correction notification mechanism must be created in order to show changes to the information. This system must allow access to the previous versions of the EHRs, if necessary.
Audits	An audit register should include all accesses to the information and all the changes that have taken place to the EHRs. This system allows the monitoring of access and is a powerful tool to guarantee a secure system. This audit system should fulfill the interoperability requirements.
Archiving	Medical records should be archived for a set period of time, according to the legislation of the respective country. After this period of time, the medical data may be deleted. However, this is not recommended when it comes to EHR management and practice, where the aim is to keep the complete medical information about the patient for his or her lifetime. However, from a logistical standpoint, this would have massive long-term storage requirements.

##### Role-Based Access

**Figure 1:**
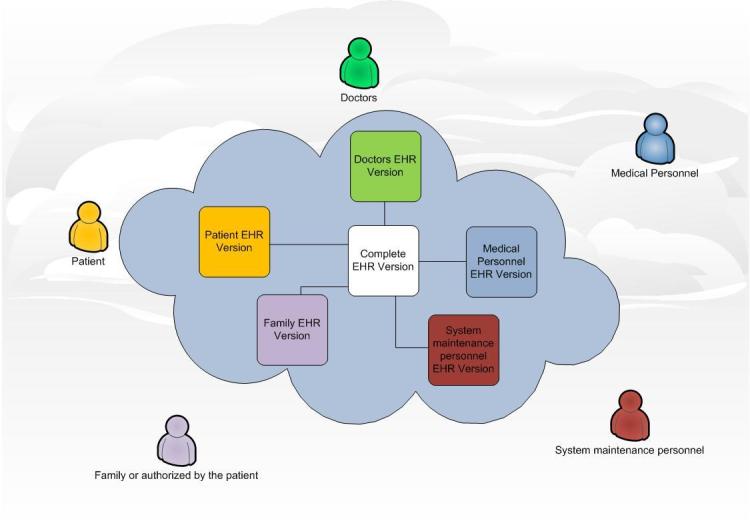
Role-based system with different electronic health record versions available depending on the kind of user of the Health Cloud.

There are many different kinds of personnel who will have access to the patient health record, from the patients themselves to the technicians responsible for the management of the provider’s servers. Physicians, medical personnel, or employees of the Cloud service provider could have access to these data. To ensure the privacy of the patient data, a role-based access system is needed because a doctor may have different access requirements to the patient information than other technical personnel. In order to overcome this problem, an ID code or number must be assigned to each person allowed to access the stored information. Depending on the ID number, the user will belong to a group and each kind of group will have access to a certain part of the patient information [22-26]. For example, patients and doctors will get access to the entire health record whereas the personnel responsible for maintenance of the platform will be able to access only the information they need for proper system operation. With this role-based system, the patients’ privacy is relatively guaranteed. Figure 1 illustrates the different roles that could take part in a Health Cloud and the different versions they will have access to.

##### Network Security Mechanisms

The main risk to the information will likely be “outside” the Cloud platform. The provider personnel are not the main threat that has to be feared. It is important to know that when moving patient data to the Cloud, health care providers are exposing this information to several external threats because the data are now available via the Internet [23]. Therefore, the responsibility must lie with the Cloud provider itself to protect the security and privacy of the information by providing the security needed to avoid external attacks to steal or even delete the information.

###### Data Encryption

All sensitive patient information must be stored securely in a private medical record so that medical information can be shared by different doctors or medical personnel. In order to secure this transaction, the information must be properly encrypted and controlled.

###### Digital Signature

The digital signature is a very useful tool that provides authenticity, integrity, and nonrepudiation [14-15]. With this security mechanism, the authenticity of the digital record is guaranteed; it will be valuable to deploy this kind of system in the Health Cloud in order to avoid false data transactions. For messages sent through an unsecure channel, the digital signature gives the receiver the reassurance that a message or file was sent by the claimed sender. There are many cryptographic logarithms to deploy this kind of security tool [23].

##### Monitoring of System Access

Every access to the platform should be monitored in order to create a log of all the people that have had access to the system. In case of an incident, the log can be consulted to solve or find out the cause of the problem. It would be valuable to create a log to track every update and change to each medical record [23].

### Suggestions Before Moving Electronic Health Records to the Cloud

The main worries of health care providers planning to move patient information to the Cloud are data security and privacy. Migrating data to the Cloud means that a third party now has control over the Cloud-hosted data. In order to address the risks that could arise, Cloud clients should be well informed before moving data to the Cloud. In order to facilitate this process, the Cloud service provider’s customers themselves should be informed about the services the Cloud provider offers them and the security mechanisms installed on the provider’s servers. Cloud clients should demand total transparency from the Cloud service provider. Knowing this kind of information is critical to being able to choose the most suitable provider for the client’s needs. Table 2 shows several security issues a client should consider when choosing the most appropriate provider [21].

### Moving Electronic Health Records to the Cloud: Example of a Cloud Company’s Security Requirements

Health care providers that decide to move their EHRs to the Cloud should be aware of these kinds of security mechanisms before migrating their records. There are several well-known Cloud service provider companies, for example, Amazon Web Services, Microsoft Cloud, GoGrid, or Salesforce, with similar security terms as explained below. Thus, this section is useful in the case of choosing a Cloud service provider. Based on the security deployed on several Cloud platforms, we suggest the following mechanisms to secure the Cloud system [22,26,27].

#### Third-Party Certification

In order to guarantee the safety of the data and meet the requirements of privacy policies, the Cloud provider must be compliant with various certifications and third-party requirements (see Table 3).

#### Monitoring

The provider should include automated monitoring tools to provide a high level of service performance and system availability. These tools should be available online for internal and external use.

Notification alarms can be configured when any modification of the data is made by the maintenance personnel or the users themselves. These tools will help track all the information changes made to the stored cloud data. Any kind of incident with the stored data will be monitored.

#### Information and Communication

In order to use the Cloud platform as a communication channel where personnel could be notified and kept up to date on everything that happens, the Cloud provider should employ various methods of internal communications in order to help employees to understand their roles and responsibilities, and to communicate significant events, if necessary. These communication methods could include orientation and training programs for newly hired personnel, video conferencing, and email, among others.

#### Employee Lifecycle

Several policies are established in the Cloud platform to manage user access. The Cloud service provider should require that staff with potential access to the patient data undergo an extensive background check (as permitted by law) commensurate with their position and level of data access. Some of these policies are shown in Table 4.

#### Physical Security

The data center building should be strictly controlled and secured with video surveillance, expert security staff, intrusion detection systems, and other electronic means. The authorized personnel should pass through authentication controls to access the data center floors.

#### Environmental Safeguards

Innovative architectural and engineering approaches should be used in database centers so as to avoid external agents that could damage them (see Table 5).

#### Configuration Management

The company should communicate all updates on both the infrastructure and the software itself, so as to minimize any impact on the customer and the service. The software updating process should be designed to avoid unintended service disruptions and maintain the integrity of service to the customer. Before updating software, these updates should be reviewed, experimented, and approved. The Cloud provider staff would manage the data center infrastructure and be responsible for the hosting management, system scalability, availability and auditing, and security management.

#### Business Continuity Management

The Cloud service provider must guarantee the availability of the service offered. In order to ensure system availability and continuity, the company should address the security issues considered in Table 6.

#### Backups

In order to guarantee the existence of the patient data stored in the Cloud, the provider should redundantly store these data. Multiple backups of these data should be stored in different data centers in various locations.

#### Storage Service Decommissioning

When a Cloud storage service comes to the end of its useful life, the provider should guarantee that data previously stored there is completely removed from its servers. Furthermore, the provider must ensure that unauthorized personnel have not copied these data.

#### Network Security

The platform itself is not the only element that should be secured by the provider. The Cloud provider must also secure the network. The network provider should guarantee significant protection against traditional network security issues, such as those summarized in Table 7.

**Table 2:** Suggestions before moving electronic health records to the Cloud.

Security issues	Description
Data security	Because a Cloud provider will have access to all the information concerning the patients, project plans, etc, it is essential to check the provider’s reputation in the market. The provider must guarantee that its clients’ information would not be misused by any unauthorized personnel. The health care provider should check for the data protection and operational integrity services offered by the provider. Moreover, it is valuable to know the geographic location of the servers where the client data would be hosted. In brief, clients should demand total transparency.
Regulatory compliance	It is important to choose providers with security certifications and are ready for external audits. It is crucial that the provider guarantee the continuity of the service in case the provider has some kind of problem. The client must ensure that the provider operates in the country where the service will be offered. Data logging and data monitoring are important tools that Cloud providers should offer in order to improve the security of the service.
User authentication	Because the data are processed externally by a third party, there is always some inherent risk. The client must know about the personnel who will manage the medical information and what standards for access will be followed by the provider. The client must be informed about the role-based access systems as well as the password handling system configured by the provider.
Data separation	The provider not only handles the data stored in the Cloud but manages the data of other companies who have hired its services. So it is important to know the mechanisms the Cloud provider implements to separate the data of all the companies that are sharing the same servers. The clients must be informed about the availability of the data that the provider guarantees.
Legal issues	A legal framework must guide the policies of the Cloud provider. Intellectual property rights agreements between the two parties should be of prime importance. While the provider owns the right to its infrastructure and applications, the client owns the right to his/her data and computational results.

**Table 3:** Third-party certifications of the Cloud provider.

Certification	Brief overview
SAS70 Type II	Statement on Auditing Standards No 70: Auditing statement that provides guidance to service auditors when assessing the internal control of a service organization and issuing a service auditor’s report.
PCI DSS Level 1	The Cloud provider should be certified with the PCI Data Security Standard as a shared hosting service provider.
ISO 27001	Certification of the Information Security Management System (ISMS) that covers infrastructure, data centers, and service terms.
FISMA	Certification to operate at Federal Information Security Management Act (FISMA) Low Level, which is a US federal law enacted in 2002. It recognizes the importance of information security to the economy and national security interests of the United States.

**Table 4:** Employee lifecycle policies of a Cloud provider platform.

Policy	Brief overview
Account provisioning	The Cloud provider itself assumes the responsibility of provisioning employees and contractor access. This access to the resources hosted in the Cloud platform must be explicitly approved by the owner or data manager.
Account review	Every access account is reviewed in Cloud platforms every 90 days.
Access removal	Every employee’s access account is automatically revoked when it is concluded.
Password policy	Access to the platform is performed by user IDs and passwords to authenticate users to services, resources, and devices, as well as to authorize the appropriate level of access to each user.

**Table 5:** Environmental safeguards installed in data centers.

Safeguard	Brief overview
Fire detection and suppression	Automatic fire detection and suppression systems are installed in the data center rooms to remove the risk of fire.
Power	24/7 electrical power systems that guarantee the uninterruptible running of the service.
Climate and temperature	In order to prevent overheating of the servers, climate control is required. This is a critical concern for the data center management and consumes lots of energy.
Management	Monitoring systems to control the state of the database equipment.

**Table 6:** Business continuity management.

Term	Brief overview
Availability	Data centers are built in clusters per regions. In case of failure of one of these data centers, automated processes move the client data traffic away from the affected area.
Incident response	Technical support and coverage to solve any kind of problem 24/7/365 (24 hours a day, 7 days a week, and 365 days a year) must be offered.
Company-wide executive review	A Cloud company should be periodically audited and supported by an internal audit group.

**Table 7:** Protection against network security issues.

Security network issue	Overview
DDoS attacks	Distributed Denial of Service (DDoS) mitigating techniques is included in the Amazon Web Services (AWS) platform to avoid this kind of attack.
MITM attacks	Man In The Middle (MITM) attacks are avoided because all the endpoints of AWS are secured by Secure Socket Layer (SSL), which provides server authentication.
IP spoofing	Traffic platform is controlled by a firewall infrastructure. Then the stored data cannot send spoofed network data.
Port scanning	Unauthorized port scans by customers are a violation of the provider’s use policy. Every reported violation should be investigated.

## Discussion

### Principal Findings

Migrating electronic health records (EHRs) to the Cloud may represent a great step in the digitalization of medical data. Advantages like scalability, economic model of pay per use, and involving the patient as an active part of the health information management process may assume a change of model in the management of medical records. Several requirements must be taken into account when the time comes to migrate sensitive and private data to the Cloud. Of those requirements, security and privacy of data are the most important ones. In storing the sensitive data of patient health records, Cloud service providers and health care providers must ensure the privacy and confidentiality of the Cloud-hosted data. In order to make this process easier, health care providers, either private or public clinical centers, that have decided to deploy this kind of system, must inform their patients of the change in how their data will be managed and stored. Additionally, a relationship of trust between the health care provider and the Cloud service provider is an essential factor in this process. In order to achieve this trust, the Cloud provider must guarantee that the security mechanisms are in place to protect the security and privacy of the stored data. An external company is needed to audit the Cloud platform provider in order to show transparency in the management information process. Legislative mechanisms regarding the security of data may be important. Comparing the security terms of several cloud computing companies will be valuable in order to choose the most suitable provider.

### Conclusion

With the emergence of Cloud computing, EHR management systems are facing an important platform shift, but such important changes must be approached carefully. In order to make a secure and smooth transition, studying all the security requirements regarding the privacy and confidentiality of patient data are essential. The Cloud computing paradigm is still under development but stands to become revolutionary in many different fields. In the near future, more services and apps will be available, and development will be enhanced.

## Abbreviations

AWS: Amazon Web Services
DDoS: Distributed Denial of Service
EHR: Electronic Health Record
FISMA: Federal Information Security Management Act
HL7: Health Level Seven
HIPAA: Health Insurance Portability and Accountability Act
ISMS: Information Security Management System
MITM: Man In The Middle
PHI: protected health information
SSL: Secure Socket Layer
